# BACE1 Inhibitor MK-8931 Alters Formation but Not Stability of Dendritic Spines

**DOI:** 10.3389/fnagi.2018.00229

**Published:** 2018-07-26

**Authors:** Tanja Blume, Severin Filser, Anna Jaworska, Jean-Francois Blain, Gerhard Koenig, Katrin Moschke, Stefan F. Lichtenthaler, Jochen Herms

**Affiliations:** ^1^Deutsches Zentrum für Neurodegenerative Erkrankungen (DZNE), Helmholtz-Gemeinschaft Deutscher Forschungszentren (HZ)—German Center for Neurodegenerative Diseases, Munich, Germany; ^2^FORUM Pharmaceuticals Inc., Waltham, MA, United States; ^3^Neuroproteomics, School of Medicine, Klinikum Rechts der Isar, Technical University of Munich, Munich, Germany; ^4^Munich Cluster for Systems Neurology (SyNergy), Munich, Germany; ^5^Zentrum für Neuropathologie und Prionforschung, Munich, Germany

**Keywords:** Alzheimer’s disease, dendritic spines, *in vivo* two-photon microscopy, BACE1 inhibition, MK-8931

## Abstract

Beta-site amyloid-precursor-protein cleaving enzyme 1 (BACE1) is the rate limiting protease in the production of the amyloid-beta peptide (Aβ), which is considered to be the causative agent in the pathogenesis of Alzheimer’s Disease (AD). Therefore, the therapeutic potential of pharmacological BACE1 inhibitors is currently tested in clinical trials for AD treatment. To ensure a positive clinical outcome it is crucial to identify and evaluate adverse effects associated with BACE1 inhibition. Preclinical studies show that chronic blockade of BACE1 activity alters synaptic functions and leads to loss of dendritic spines. To assess the mechanism of synapse loss, dendritic spine dynamics of pyramidal layer V cells were monitored by *in vivo* two-photon microscopy in the somatosensory cortex of mice, treated with the BACE1 inhibitor MK-8931. MK-8931 treatment significantly reduced levels of Aβ40 and density of dendritic spines in the brain. However, the steady decline in dendritic spine density specifically resulted from a diminished formation of new spines and not from a loss of stable spines. Furthermore, the described effects on spine formation were transient and recovered after inhibitor withdrawal. Since MK-8931 inhibition did not completely abolish spine formation, our findings suggest that carefully dosed inhibitors might be therapeutically effective without affecting the structural integrity of excitatory synapses if given at an early disease stage.

## Introduction

Beta-site amyloid-precursor-protein cleaving enzyme 1 (BACE1) is considered as a potential drug target for therapeutic intervention in Alzheimer’s disease (AD) for years. According to the amyloid-cascade-hypothesis the molecular cause of AD is the pathological accumulation and aggregation of the amyloid-beta peptide (Aβ). BACE1 is considered to be the rate determining step in Aβ production by initiating the amyloidogenic pathway trough cleavage of the amyloid precursor protein (APP), which is followed by γ-secretase cleavage (Hardy and Higgins, 1992; Selkoe, 2002; Selkoe and Hardy, 2016). Accordingly, it has been shown that BACE1 inhibition efficiently lowers Aβ levels in the central nervous system of healthy subjects and AD patients (Forman et al., 2012; Neumann et al., 2015; Thakker et al., 2015; Kennedy et al., 2016). These encouraging findings led to further evaluation of MK-8931 (Verubecestat), a BACE1 inhibitor with good oral bioavailability and blood-brain-barrier permeability (Forman et al., 2013).

However, the physiological role of APP cleaving enzymes in the mature nervous system is incompletely understood and has to be investigated in more detail. This lack of knowledge might have contributed to the failure of the γ-secretase inhibitor LY-450139 (Semagacestat) in clinical trials. Prolonged treatment with LY-450139 caused worsening of cognitive functions in AD patients as well as infections and skin cancer (Schor, 2011) leading to early termination of the study in clinical phase III, April 2011 (Doody et al., 2013). Altered cognitive function during long-term γ-secretase inhibition in humans might be associated with the preclinical observation that treatment with LY-450139 causes a persistent reduction in dendritic spine density in mice (Bittner et al., 2009).

Dendritic spines are highly plastic structures which are continuously formed and eliminated throughout life. According to their lifetime dendritic spines can be distinguished into two classes: Transient spines with a lifetime of less than 4 days and stable spines which persist more than 8 days (Holtmaat et al., 2005). The structural dynamics of dendritic spines represent a central cellular mechanism in the formation of memory (Chklovskii et al., 2004; Holtmaat and Svoboda, 2009; Xu et al., 2009; Yang et al., 2009). Short-lived transient spines are associated with learning (Bourne and Harris, 2008), whereas stable spines are associated with long-term memory (Yang et al., 2009). Therefore, the worsening of memory observed in the LY-450139 trial might be attributable in part to the loss of dendritic spines.

Importantly, it has been shown recently that prolonged treatment with BACE1 inhibitors also reduces dendritic spine densities in wildtype mice (Filser et al., 2015; Zhu et al., 2018). However, differential inhibitor effects on transient and stable spine populations have not been analyzed, although this information would provide clear implications for the interpretation of prospective clinical outcomes.

To decipher the cause of BACE1 inhibitor induced spine loss, we first treated mice chronically with the clinically most advanced BACE1 inhibitor MK-8931 and confirmed inhibitor efficacy by determining cortical Aβ levels via sandwich ELISA. To clarify whether BACE1 inhibition leads to a loss of stable spines or to a decrease in spine formation, we performed long-term *in vivo* two-photon imaging of transgenic mice expressing eGFP in cortical layer V pyramidal neurons and analyzed the kinetics of spine turn-over and stability.

As anticipated, our data showed that BACE1 plays an important role in the regulation of structural spine plasticity. In particular, we observed a significant but reversible BACE1 inhibitor effect on spine formation, whereas a loss of stable spines was not detected. Thus, pharmacological BACE1 inhibition specifically altered dendritic spine formation but did not disrupt substantially the structural integrity of excitatory synapses.

## Materials and Methods

### BACE1 Inhibitor

The BACE1 inhibitor MK-8931 was synthesized following the schemes provided by FORUM Pharmaceuticals (Waltham, MA, USA) and formulated in 10% (w/v) 2-hydroxypropyl-beta-cyclodextrin. MK-8931 was administered at 20 mg/kg body weight by oral gavage every 12 h for 21 days.

### Animals

Female and male 3 month old heterozygous GFP-M mice (Tg(Thy1-EGFP)MJrs from Jackson Laboratory) were used (Feng et al., 2000). Mice were group-housed under pathogen-free conditions and bred in the animal housing facility at the Center for Neuropathology and Prion Research of the Ludwig-Maximilians-University of Munich, with food and water provided *ad libitum* (21 ± 1°C, at 12/12 h light/dark cycle). After cranial window implantation, mice were housed separately. All applicable international, national and/or institutional guidelines for the care and use of animals were followed. All experiments were carried out in compliance with the National Guidelines for Animal Protection, Germany with the approval of the regional Animal care committee of the Government of Oberbayern (Regierung Oberbayern) and were overseen by a veterinarian. Animal experiments were conducted in accordance with the guidelines EU Directive 2010/63/EU for animal experiments.

### Aβ Purification and ELISA Assay

Cortices from C57BL/6J mice were isolated 4 h after the last drug administration, frozen and homogenized in 1 ml of 2% diethylamine, containing 50 mmol/L sodium chloride (pH 10), complete protease inhibitors (Sigma-Aldrich, St. Louis, MO, USA), and 2 mmol/L EDTA. Samples were homogenized with a rotor-stator homogenizer, directly followed by neutralization of the homogenate with Tris-HCl buffer, pH 6.8. Suspensions were then centrifuged at 3500× *g* at 4°C for 5 min. Supernatants were transferred to new vials and subjected to clarifying ultracentrifugation at 130,000× *g* at 4°C for 30 min to obtain the mass fraction of diethanolamine (DEA) fraction. Pellets from the first centrifugation were washed with PBS to remove the remaining soluble fraction and then lysed in STE buffer (150 mM NaCl, 50 mM Tris-HCl, 2 mM EDTA) and 1% Triton X-100 for 60 min on ice followed by centrifugation at 20,000× *g* for 10 min at 4°C. The resulting insoluble fractions were transferred to fresh tubes. Protein concentration of insoluble or DEA fractions was measured using the (BCA) method according to the manufacturer’s instructions (Interchim, Mannheim, Germany). Aβ40 levels of both tissue fractions were measured using MSD Aβ triplex sandwich immunoassay (MesoScale Discovery, Rockville, MD, USA) in accordance with the protocols provided by the manufacturer. The concentrations of Aβ40 peptide were calculated using the MSD discovery workbench software and normalized to protein content. Combined total normalized Aβ40 levels of brain homogenates are displayed in the “Results” section.

### Cranial Window Implantation

Mice were anesthetized with an intraperitoneal injection of ketamine/xylazine (130/10 mg/kg body weight; WDT/Bayer Health Care) and dexamethasone (6 mg/kg body weight, Sigma) was applied to prevent development of cerebral edema. A circular piece of the skull (3 mm in diameter) over the somatosensory cortex (coordinates of craniotomy: Bregma +1.5 to −3.5 mm, 3 mm lateral from midline) was removed and replaced with a cranial window, as described before (Fuhrmann et al., 2007; Holtmaat et al., 2009). In addition, a metal bar was attached to the skull to allow repositioning of the mouse during subsequent imaging sessions. After surgery, mice received subcutaneously antibiotic Cefotaxim (2.5 mg/kg body weight; Pharmore) and Rimadyl (7.5 mg/kg body weight).

### *In Vivo* Two-Photon Imaging

After a recovery period of 4 weeks, mice were imaged with a LSM 7MP microscope (Zeiss) equipped with a water-immersion objective (20×, NA = 1.0; Zeiss). Mice were anesthetized with isoflurane (1%–1.2% in 95% O_2_ and 5% CO_2_), placed on a heating pad and fixed to a custom-made holder using the attached metal bar. eGFP was excited with a femtosecond laser (Mai Tai DeepSee, Spectra Physics) at a wavelength of 920 nm. Apical dendritic tufts of layer V pyramidal neurons in the somatosensory cortex were imaged in consecutive sessions every week. Three imaging positions per animal were chosen, with up to ten dendrites per region of interest. Each imaging session did not last more than 60 min. The same imaging position was retrieved based on the vascular pattern of the cortex and the stable branch points of apical dendritic shafts. For overview images, z-stacks of 300 μm depth with 3 μm axial resolution and 1024 × 1024 pixels per image frame (0.4 μm/pixel) were acquired. For dendritic spines, high-resolution images were taken with 1 μm axial resolution and 512 × 256 pixels per image frame (0.1 μm/pixel). To keep the emitted fluorescence stable at different depths and also at subsequent imaging sessions, the z-correction tool in the microscope control software was used, which allows an adjustment of the laser intensity.

### Analysis of Dendritic Spine Plasticity

Dendritic spines were counted manually in ZEN 2011 (Zeiss). Due to small movements artifacts of the tissue e.g., because of pulsation of nearby capillaries, images were analyzed in three dimensions and not in z-projections. All clear spines emanating laterally from the dendritic shaft, regardless of apparent shape, with a length of >0.4 μm (Holtmaat et al., 2009) were measured. Spines were counted as stable when they were detected at the first time point of the imaging period, as well as a week later at the same place, not >0.5 μm away from the previous position. Lost spines were counted as lost when they consisted of <0.4 μm length and as gained when they consisted of >0.4 μm in length protruding from the dendrite. This scoring method has already been used successfully (Holtmaat et al., 2005; Fuhrmann et al., 2007). Because of resolution limitations in the z-plane, only spines emanating from the dendrite in the x-y direction were counted. As defined by Holtmaat et al. (2005), spines are classified as transient if they last less than 4 days. However, in the current study dendritic spine kinetics were evaluated on a weekly basis. With respect to the longer imaging interval we thus considered spines with a lifetime of less than 7 days as transient. Daily turnover rate, representing the gain and loss of spines from day to day, was calculated as following: (N_gained_ + N_lost_)/(2 × N_total_)/I_t_, where N_gained_ is the number of gained spines, N_lost_ is the number of lost spines, N_total_ is the number of all apparent spines at a time point, and I_t_ is the number of days between subsequent imaging sessions (Holtmaat et al., 2005; Fuhrmann et al., 2007). Five animals per group and ten dendritic segments of approximately 22–35 μm length were analyzed per mouse.

### Statistics

Statistics were calculated in GraphPad Prism 5.04 (GraphPad Software, San Diego, CA, USA). Intergroup comparisons were performed using two-tailed Student’s *t*-test. Dendritic spine dynamics from day 1 until the end of the treatment (day 0–28) were compared using two-way ANOVA treatment main factor with Bonferroni *post-hoc* test. All results were normalized and are presented as mean ± SEM. Column factor *p* values < 0.05 are defined as statistically significant.

## Results

### MK-8931 Treatment Reduces Aβ40 Levels in Mouse Cortex

Preclinical data has shown that the orally administered BACE1 inhibitor MK-8931, crosses the blood-brain barrier and remains stable in the brain up to 12 h in rats (Forman et al., 2012; Kennedy et al., 2016). Chronic treatment with 12 and 40 mg/kg MK-8931 resulted in a dose-dependent reduction in cerebrospinal fluid (CSF) Aβ levels after 12 h, of 40% and 80% respectively, in mild-to-moderate AD patients (Forman et al., 2013; Andrew Stamford, 2015) and in the transgenic AD mouse model Tg2576 (Villarreal et al., 2017).

**Figure 1:**
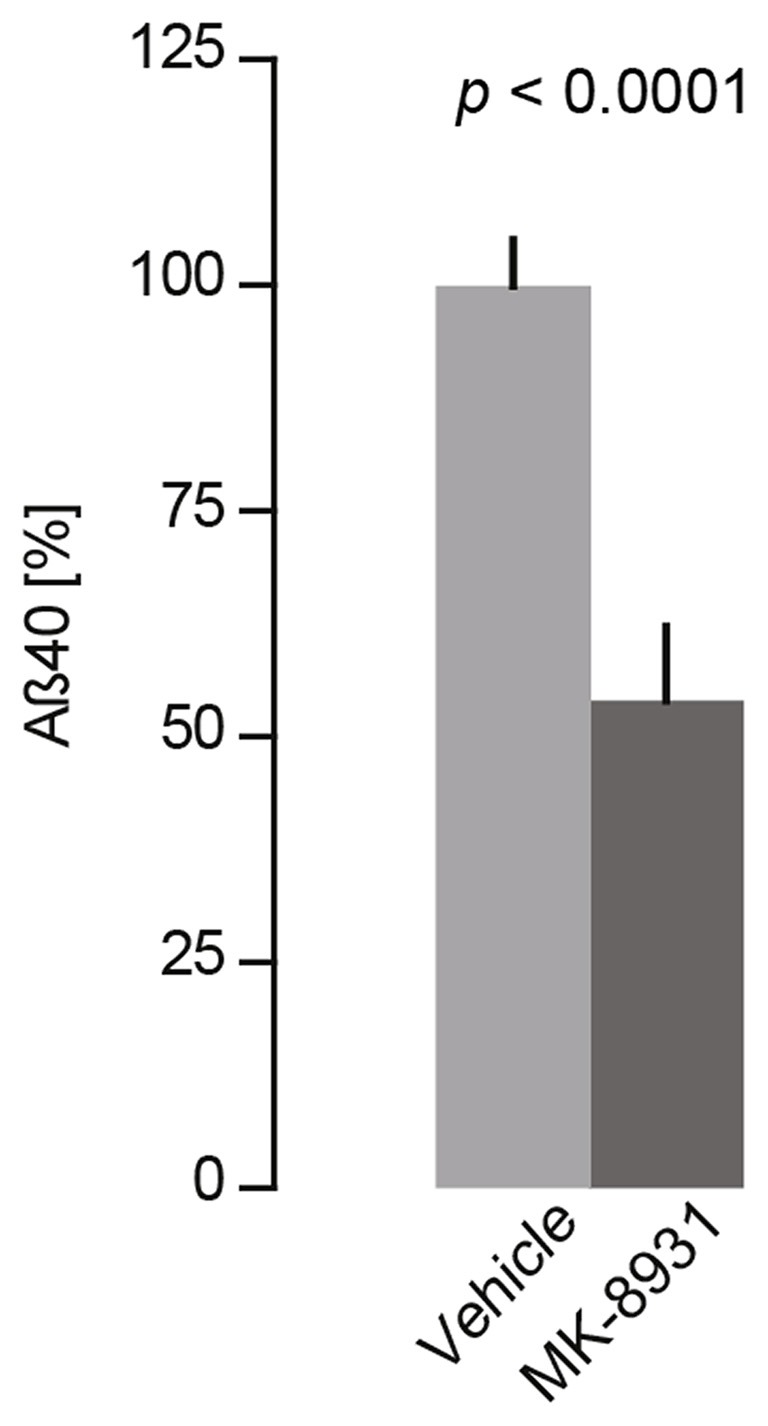
MK-8931 (20 mg/kg) treatment significantly reduced Aβ 40 levels in the murine cortex (*t*_(6)_ = 9.5, *p* < 0.0001, two-tailed unpaired Student’s *t*-test). Brain samples were taken 4 h after acute treatment and were analyzed by Sandwich-ELISA assay. Data presented as mean ± SEM, *n* = 4.

To verify these findings, we measured acute Aβ40 levels in the cerebral cortex in four C57BL/6J wildtype mice after 4 h of treatment with a single dose of 20 mg/kg of MK-8931 or vehicle. ELISA measurements of brain homogenates showed that MK-8931 treatment reduced Aβ40 levels by 47% ± 4% compared to vehicle treatment (*t*_(6)_ = 9.5, *p* < 0.0001, unpaired *t*-test, Figure 1).

### MK-8931 Treatment Alters Dendritic Spine Formation

**Figure 2:**
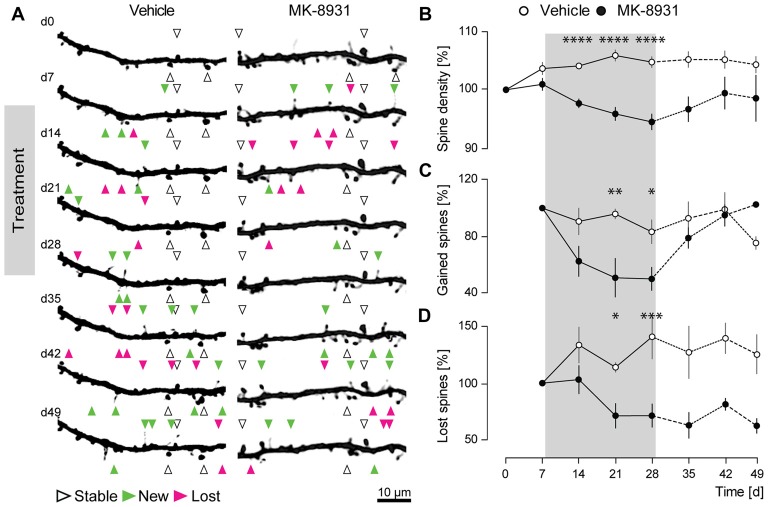
BACE1 inhibitor MK-8931 altered plasticity of dendritic spines *in vivo*. **(A)** Micrographs of eGFP-labeled apical dendrites of layer V pyramidal neurons in somatosensory cortex before, during and after administration of vehicle or MK-8931. Treatment started 8 days after first imaging timepoint and was continued over 21 days, every 12 h. White arrowheads exemplarily mark representative spines which were stable over the entire imaging period. Newly gained spines are labeled with green arrowheads and lost spines are labeled with magenta arrowheads. **(B)** Quantification of spine density **(C,D)** fraction of gained and lost spines in mice treated with vehicle or MK-8931 (20 mg/kg). *N* = 5 animals per group, *n* = 10 dendrites per animal. Data is presented as mean ± SEM. Bonferroni *post-hoc* test results: *p < 0.05, **p < 0.01, ***p < 0.001, ****p < 0.0001 from two-way ANOVA (day 0–28).

Prior studies with two different BACE1 inhibitors showed a significant decrease in the fraction of newly gained spines, implicating an important role for BACE1 in promoting dendritic spine development (Filser et al., 2015). To investigate, if similar effects could be observed with the clinically most advanced BACE1 inhibitor MK-8931, we recorded the structural dynamics of apical dendritic spines of layer V pyramidal cells in the somatosensory cortex of adult GFP-M mice treated with MK-8931, by chronic *in vivo* two-photon microscopy (Figures 2A,B). The treatment period with MK-8931 (20 mg/kg, every 12 h) as well as vehicle lasted for 21 days.

As anticipated, we observed a significant decrease of 6% ± 1.4% (*F*_(1,8)_ = 59.36, *p* < 0.0001, ANOVA treatment main factor, Figure 2B) in total spine density after 21 days of MK-8931 treatment. Furthermore, MK-8931 treatment strongly affected the formation of dendritic spines. The fraction of new spines decreased by 50% ± 8.2% (*F*_(1,8)_ = 11.02, *p* = 0.0105, ANOVA treatment main factor) after 21 days of BACE1 inhibitor treatment compared to the vehicle group (Figure 2C). BACE1 inhibition also decreased significantly the fraction of lost spines, although to a smaller degree, by 29% ± 10.3% (*F*_(1,8)_ = 10.52, *p* = 0.0118, ANOVA treatment main factor, Figure 2D).

These observations confirmed a critical function of BACE1 in the regulation of dendritic spine formation.

### MK-8931 Treatment Does Not Cause Spine Elimination

Next, we investigated whether BACE1 inhibition has an effect on spine stability in the somatosensory cortex.

**Figure 3:**
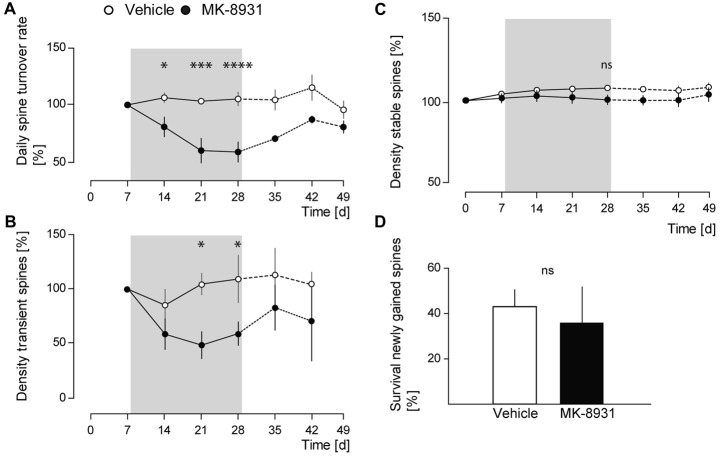
MK-8931 treatment did not cause spine elimination. **(A)** Spine stability and turnover rates in vehicle and MK-8931 treated mice. Daily turnover rate significantly decreased at the end of inhibitor treatment. **(B,C)** MK-8931 treatment significantly decreased density of transient spines, whereas density of stable spines was not affected. Bonferroni *post-hoc* test: ns: *p* = 0.4926, *p < 0.05, ***p < 0.001, ****p < 0.0001 (two-way ANOVA between day 0–28). **(D)** Survival rate of newly gained spines was not affected by BACE1 inhibitor treatment. *N* = 5 animals per group, *n* = 10 dendrites per animal. Two-tailed Student’s *t*-test, *t*_(8)_ = 0.9, *p* = 0.394. Data presented as mean ± SEM.

We analyzed daily spine turnover rates in MK-8931 treated mice and detected a significant decrease by 42% ± 8.8% (*F*_(1,8)_ = 17.05, *p* = 0.0033, ANOVA treatment main factor, Figure 3A). To analyze the decrease in spine turnover in more detail, we quantified the density of transient and stable spines before, during and after the treatment period. No significant effect on stable spine density was detected (*F*_(1,8)_ = 2.14, *p* = 0.1817, ANOVA treatment main factor, Figure 3C), whereas the density of transient spines decreased significantly by 41% ± 11.5% (*F*_(1,8)_ = 7.38, *p* = 0.0264, ANOVA treatment main factor, Figure 3B).

Furthermore, we analyzed the survival rate of newly gained spines. We observed an apparent but statistically non-significant decrease in MK-8931 treated mice (*t*_(8)_ = 0.9, *p* = 0.395, unpaired Student’s *t*-test, Figure 3D). After termination of BACE1 inhibitor treatment we recognized a recovery in spine density, gained and transient spines, as well as in daily spine turnover rate. These parameters did not differ significantly between both cohorts at imaging day 42, while the fraction of lost spines remained significantly reduced in the inhibitor treated group (*p* = 0.015, unpaired Student’s *t*-test). In summary, our data showed that BACE1 inhibition specifically altered dendritic spine formation. Thus, the reduction of dendritic spine density in MK-8931 treated mice was caused by diminished formation of new spines, whereas spine stability was not affected.

## Discussion

In the present study, we demonstrated that chronic treatment with the BACE1 Inhibitor MK-8931 reduced total dendritic spine density of layer V pyramidal cells in the somatosensory cortex of adult mice. To unravel the mechanistic cause of this phenotype we applied intravital microscopy and analyzed the kinetics of structural spine plasticity. Our data showed that MK-8931 treatment specifically reduced the formation rate of new dendritic spines in a reversible manner, but did not cause an elimination of stable spines. Therefore, MK-8931 treatment does not alter the overall structural integrity of excitatory synapses.

In contrast, γ-secretase inhibitor application has been shown to cause elimination of stable spines with a persistent reduction in spine density *in vivo* (Bittner et al., 2009). Furthermore, genetic ablation of the γ-secretase substrate Notch1 in pyramidal CA1 neurons alters spine density as well as spine morphology and leads to deficits in learning and short-term memory (Alberi et al., 2011). Accordingly, treatment with the γ-secretase inhibitor LY-450139 led to worsening of cognitive functions in AD patients and thus failed in clinical phase III trials (Schor, 2011; Doody et al., 2013; Funded by Eli Lilly, ClinicalTrials.gov, number: NCT00594568).

The mechanism-based side effects of BACE1 inhibition on dendritic spines are thus more subtle compared to γ-secretase inhibition. However, although the most promising BACE1 inhibitor MK-8931 effectively lowered Aβ CSF levels in healthy adults and showed no obvious side effects (Forman et al., 2013), it failed in phase III clinical trials. The treatment with MK-8931 failed to significantly slow down the disease progression in mild to moderate AD patients and also had no positive effect in prodromal AD patients (Hawkes, 2017; Egan et al., 2018). These observations suggest that BACE1 inhibitor treatment should begin even earlier, preferably at a stage when individuals are still cognitively normal, to provide clinical benefit. This claim is supported by a recently published *in vivo* animal study showing that BACE1 inhibitor treatment does not completely stop the growth of established plaques but strongly prevents the formation of new plaques (Peters et al., 2018).

Thus, early pharmacological intervention in a timely manner seems more promising to halt the pathological cascade leading to AD. However, although short-term BACE1 inhibitor treatment seems to be well tolerated, even subtle side effects might culminate in a detrimental outcome if BACE1 inhibition persists over many years. In this context it has to be considered that Aβ, at physiological levels, plays an essential role in synaptic plasticity and memory (Dawson et al., 1999; López-Toledano and Shelanski, 2004; Laird et al., 2005; Zou et al., 2016). In particular Puzzo et al. (2008) showed, that application of physiological doses of Aβ42 monomers and oligomers evokes a significant increase in synaptic long-term potentiation, whereas high Aβ levels reduce hippocampal long-term potentiation. Furthermore, BACE1 is involved in the proteolytic processing of the transmembrane protein neuregulin 1 (Nrg1). The N-terminal cleavage products of Nrg1 activates receptor tyrosine kinase ErbB dependent signaling pathways with numerous roles in CNS development and functions, like synapse formation, plasticity and axon myelination (Falls, 2003; Michailov et al., 2004; Savonenko et al., 2008). Therefore, it will be necessary to determine the optimal inhibitor dosage to avoid side effects on dendritic spines and thus balance clinical safety and efficacy.

Indeed, BACE1 inhibitor induced effects on dendritic spine formation have been shown to be dose dependent. While low- and high-dosed inhibitors both significantly reduce Aβ levels in murine brains, application of low-dosed inhibitor has no effect on structural dendritic spine plasticity (Filser et al., 2015). Interestingly, it was recently shown that pharmacological BACE1 inhibition reduces dendritic spine density via Seizure Protein 6 (Sez6; Zhu et al., 2018). Sez6 is exclusively cleaved by BACE1 and required for normal dendritic arborization as well as synaptic plasticity (Gunnersen et al., 2007; Pigoni et al., 2016). Since soluble Sez6 is detectable in CSF (Khoonsari et al., 2016), it might serve as a biomarker to evaluate the BACE1 inhibitor dosage, which is sufficient to preserve the synaptic function of BACE1.

In summary, our results show that the BACE1 inhibitor induced reduction in dendritic spine density is not caused by the elimination of existing spines, but by a reduced spine formation, which is reversible after cessation of BACE1 inhibitor treatment. Therefore, the mechanism-based side effects of BACE1 inhibition on synaptic function are less severe compared to γ-secretase inhibition and could be potentially avoided by monitoring Sez6 in CSF, which is exclusively cleaved by BACE1, to evaluate the optimal inhibitor dosage.

## Data Availability

The raw data supporting the conclusions of this manuscript will be made available by the authors, without undue reservation, to any qualified researcher.

## Author Contributions

TB, SF and AJ performed the measurements. TB and SF analyzed and quantified the data. J-FB and GK contributed the BACE1 inhibitor NB-360. KM performed measurements of soluble Aβ. TB, SF, SL and JH interpreted the data. JH, SF and TB contributed to the conception and design of the study. TB and SF wrote the manuscript. All authors approved the final manuscript.

## Conflict of Interest Statement

The authors declare that the research was conducted in the absence of any commercial or financial relationships that could be construed as a potential conflict of interest.
